# Preconditioning is an effective strategy for improving the efficiency of mesenchymal stem cells in kidney transplantation

**DOI:** 10.1186/s13287-020-01721-8

**Published:** 2020-05-24

**Authors:** Lingfei Zhao, Chenxia Hu, Fei Han, Fanghao Cai, Junni Wang, Jianghua Chen

**Affiliations:** 1 Kidney Disease Center, the First Affiliated Hospital, College of Medicine, Zhejiang University, Hangzhou, Zhejiang People’s Republic of China; 2 Key Laboratory of Kidney Disease Prevention and Control Technology, Hangzhou, Zhejiang Province People’s Republic of China; 3 Institute of Nephrology, Zhejiang University, Hangzhou, Zhejiang People’s Republic of China; 4 State Key Laboratory for Diagnosis and Treatment of Infectious Diseases, the First Affiliated Hospital, College of Medicine, Zhejiang University, Hangzhou, Zhejiang People’s Republic of China

**Keywords:** Mesenchymal stem cells, Preconditioning, Kidney transplantation

## Abstract

The inevitable side effects caused by lifelong immunosuppressive agents in kidney transplantation patients spurred the exploration of novel immunosuppressive strategies with definite curative effects and minimal adverse effects. Mesenchymal stem cells (MSCs) have become a promising candidate due to their role in modulating the immune system. Encouraging results obtained from experimental models have promoted the translation of this strategy into clinical settings. However, the demonstration of only marginal or transient benefits by several recent clinical controlled studies has made physicians hesitant to adopt the routine utilization of this procedure in clinical settings. Impaired MSC function after infusion in vivo was thought to be the main reason for their limited effects. For this reason, some preconditioning methods were developed. In this review, we aim to outline the current understanding of the preconditioning methods being explored as a strategy to improve the therapeutic effects of MSCs in kidney transplantation and promote its clinical translation.

## Introduction

Kidney transplantation is still the best treatment for end-stage renal disease (ESRD). Prevention of graft rejection and prolonged acceptance of transplanted organs are important topics in this field. Due to the development of triple immunosuppressive therapy (corticosteroids, calcineurin inhibitors (CNIs), and antimetabolites) in the last few decades, the fear of acute rejection (AR) has been greatly reduced. However, the lifelong use of combined but not specific immunosuppressants will inevitably cause various side effects, such as cardiovascular disease, metabolic complications, nephrotoxicity, and sometimes even life-threatening infections and malignancies [1]. As such, there is an urgent need to explore novel regimens to reduce or even replace lifelong immunosuppressive drug use.

Mesenchymal stem cells (MSCs) are a type of fibroblast-like multipotent cell that can be differentiated into several mesenchymal cells (e.g., osteoblasts, adipocytes, and chondrocytes). The lack of expression of major histocompatibility complex (MHC) class II or costimulatory molecules such as CD40, CD80, and CD86 and the low level of MHC-I on their surface make MSCs an ideal injected cell product with little immunogenicity. Moreover, many studies have confirmed the compelling evidence that these cells have immunoregulatory properties and can suppress the activation of multiple immune cells [2, 3]. The immunoprivileged and immunoregulatory characteristics of MSCs are promising for treatments for autoimmune diseases. For example, MSCs were found to be protective in experimental autoimmune encephalomyelitis, arthritis, type 1 diabetes, etc. [4–6] The clinical utilization of MSCs has also revealed their preliminary efficacy in graft-versus-host disease patients [7]. Furthermore, MSCs can display tissue repair effects. Attributed to their migratory, differentiative, and secretory capacity, ischemia/reperfusion (I/R) injury, which is a common situation seen during kidney transplantation, can be largely ameliorated [8]. These facts make MSCs a promising cell treatment candidate with the capacity to repair cell injury, prevent tissue rejection, and achieve organ tolerance, all of which are key factors in kidney transplantation.

In a broad range of animal kidney transplantation models, MSCs have shown potential tolerance-inducing effects. By enhancing the generation of regulatory T cells (Tregs) and targeting antigen-presenting cells (APCs), MSCs promoted donor-specific tolerance [9]. However, when they were translated into clinical settings, challenges emerged. Several recent clinical controlled studies demonstrated only a marginal or transient beneficial outcome from MSCs in kidney transplantation patients [10–13]. These contradictory results make decision-making difficult for physicians.

One explanation for the contradictory results between animal models and clinical practices is the impaired MSC functions in host tissues. During ex vivo expansion, MSCs present with reduced pluripotency as well as decreased expression of homing receptors [14]. Moreover, after infusion, MSCs suffer a harsh microenvironment in vivo, which results in poor survival and poor engraftment into the target tissues. It has been demonstrated that most intravenously infused MSCs are trapped in the liver, lungs, and spleen, and over 90% of cells die within a week [15, 16]. These challenges have weakened the idea that MSCs can serve as regulators in the balance between effector and regulatory pathways in transplantations.

**Fig. 1:**
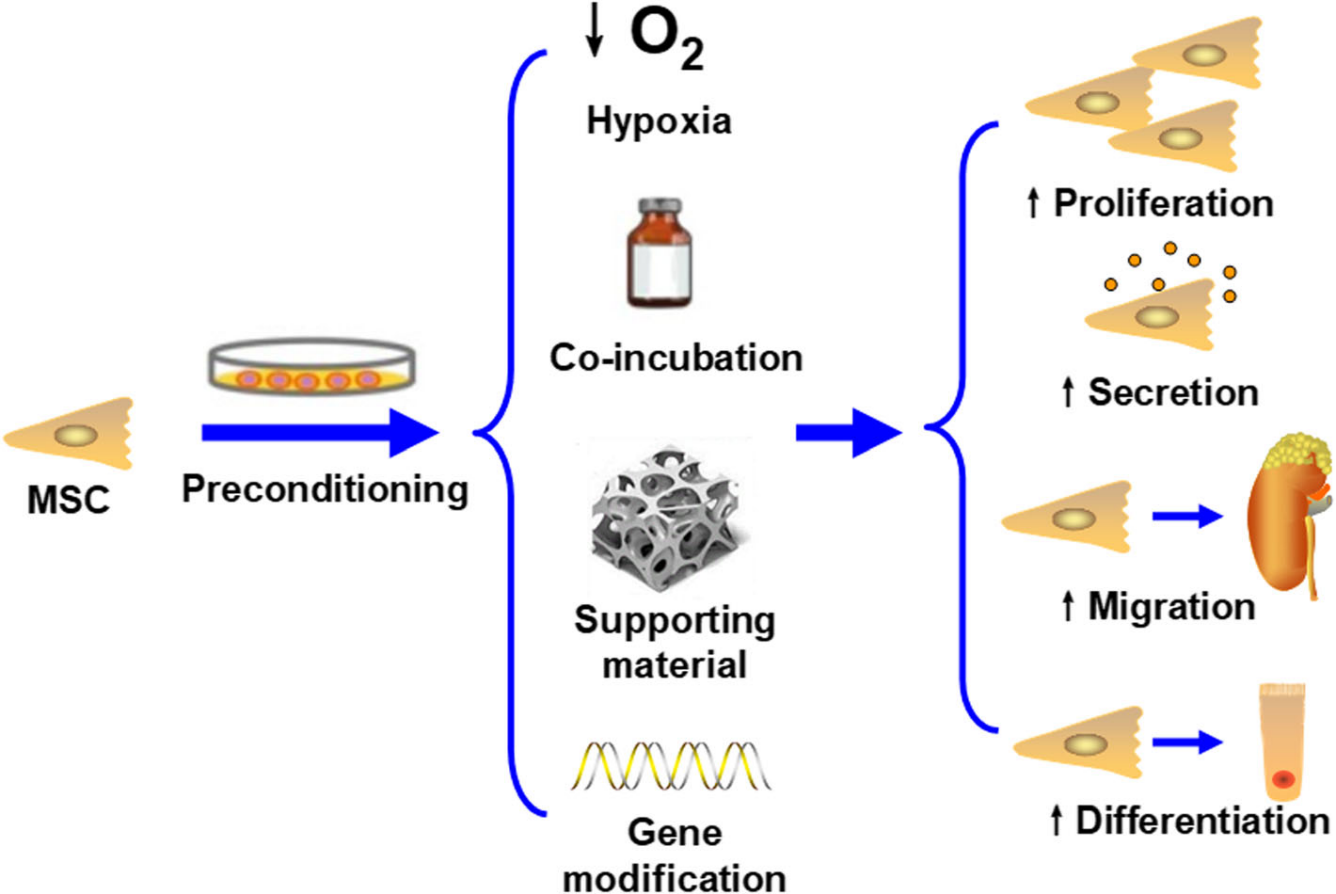
Various preconditioning methods are able to increase the proliferative, secretory, migratory, and differentiation abilities of MSCs, improving their therapeutic effects

In light of this, researchers have sought different strategies to improve the therapeutic effects of MSCs. Among them, preconditioning has generated much interest. Preconditioning is a strategy that relies on a variety of techniques to enhance the capacity of substances during ex vivo expansion. Generally, available preconditioning methods include hypoxia, incubation with pharmacological/chemical agents or trophic factors/cytokines, physical factor preconditioning, and gene modification. By proper pretreatment of MSCs before infusion, their proliferative, secretory, migratory, and differentiated abilities can be greatly improved, favoring beneficial outcomes in vivo (Fig. 1) [17].

In this review, we begin with the encouraging results in animal models. Then, challenges met in clinical controlled studies are mentioned. Finally, we list promising studies featuring preconditioning strategies for MSCs. By summarizing the available evidence, we intend to provide an integrated and comprehensive view of the best way to enhance the therapeutic effects of MSCs in kidney transplantation and improve the prognosis of ESRD patients via this regenerative medicine strategy.

## Encouraging results of MSC application in animal kidney transplantation models

**Fig. 2:**
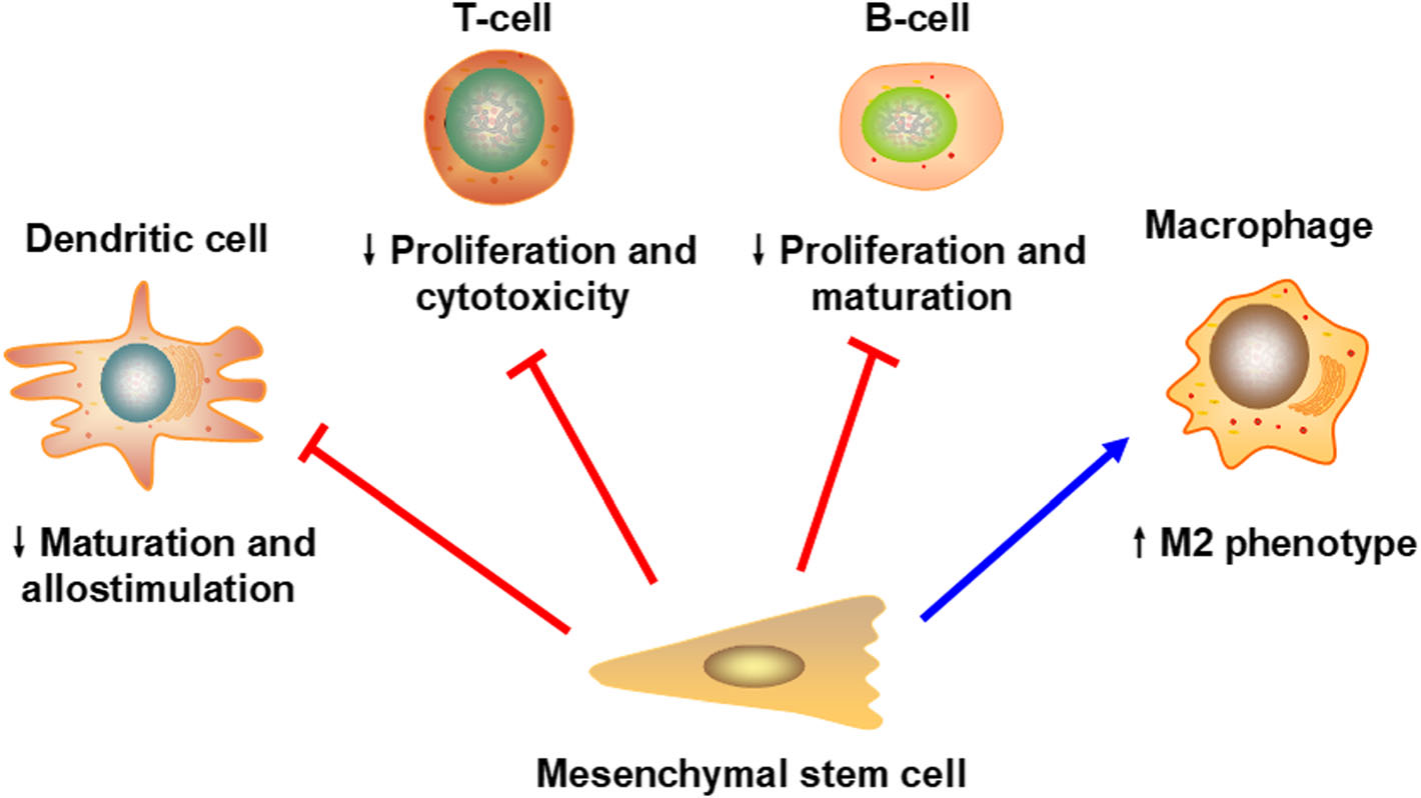
The immunomodulatory effects of MSCs in kidney transplantation. MSCs can interact with various immune cells and affect their functions. For example, MSCs can inhibit the maturation and allostimulatory function of DCs and hamper the proliferation and cytotoxicity of T cells. In addition, impaired proliferation and maturation of B cells can also be observed after incubation with MSCs. Macrophages experience a switch towards the M2 phenotype when cocultured with MSCs

In animal kidney transplantation models, MSCs have shown encouraging results. MSCs have shown immunoregulatory activities on multiple immune cells, including T cells, B cells, dendritic cells (DCs), and natural killer cells (NK cells) (Fig. 2). In detail, MSCs interfere with the maturation and antigen-presenting function of DCs [18], inhibit T cell proliferation and induce anergy [19], decrease CD8^+^ T cell cytotoxicity [20], switch T cells, and differentiate macrophages towards an anti-inflammatory phenotype [21, 22]. In addition, the cytotoxicity of NK cells could also be suppressed by MSCs [23]. In B cells, there are also data confirming the ability of MSCs to arrest B cells in the cell cycle and reduce their antibody secretion [24, 25].

By interacting with almost all immune cells of both the innate and adaptive immune systems, MSCs are thought to be able to dampen immune-mediated reactions and achieve tolerance in kidney transplantation (Table 1). Ge et al. found that transplantation of MSCs significantly prolonged the survival of allografts from 31 days to over 100 days in a mouse model of allogeneic kidney transplantation. Histologically, the transplanted group presented with minimal alterations, whereas extensive inflammatory cell infiltration, interstitial edema, and glomerular/tubular necrosis were found in the control group, which are typical signs of AR. To take a deeper look into the mechanism underlying the protective effects of MSCs, the researchers identified T cells, DCs, and Tregs in two groups of recipients. Their data revealed that the maturation of DCs and the proliferation of T cells were significantly dampened in tolerant recipients; however, the percentage of CD4^+^CD25^+^Foxp3^+^ T cells were increased in these recipients. Moreover, after blockade of the indoleamine 2,3-dioxygenase (IDO) pathway by the IDO inhibitor 1-methyl-tryptophan or transplantation of IDO-knockout MSCs (IDO^−^/^−^ MSCs), all of the abovementioned beneficial effects disappeared [26]. Similarly, Koch et al. harvested transplanted kidneys from mice that were given MSC treatment after kidney transplantation. Less T and B cell infiltration was identified in mice who received MSC treatment than in those that did not, indicating a beneficial impact of MSC administration on preventing allograft rejection [27].

**Table 1:** Encouraging results of MSCs application in animal kidney transplantation models

Year	Animal	MSCs source	Timing of infusion	Outcomes	References
2010	Mice	BM-MSCs	Day 1	↑IDO, ↑kyneurenine, ↓DCs and T cells, ↑Tregs, ↓AR, ↑graft renal function and survival	Ge et al. [26]
2013	Rats	BM-MSCs	Day 0	↓T and B cells, ↓AR, ↑graft renal function	Koch et al. [27]
2017	Mice	BM-MSCs-MVs	Day 1	↑miR-146a, ↓DCs, ↑graft renal function and survival	Wu et al. [28]
2017	Rats	BM-MSCs	Two injections (day − 7, 0 or day − 4, 0)	↓T, B, and NK cells, ↑Tregs, ↓graft inflammation, ↑graft renal function and survival	Merino et al. [29]
2017	Rats	BM-MSCs	Three injections (day 0, 3, 7)	↓AR, ↓pathological score, ↓TGF-β1	Yu et al. [30]
2007	Rats	BM-MSCs	Four injections (day − 7, 0, 7, 14)	↓AR, ↑graft renal function and survival	Zhang et al. [31]
2012	Mice	BM-MSCs	Day − 7 or day − 1 or day − 7, − 1	↑Tregs, ↓AR, ↑graft renal function and survival	Casiraghi et al. [32]
2010	Rats	BM-MSCs	Day 0	↓AR, ↓pathological score, ↑graft renal function	Zonta et al. [33]
2012	Rats	BM-MSCs	Day 77	↑IDO, ↓IF/TA, ↓graft inflammation, ↑graft renal function	Franquesa et al. [34]
2014	Porcine	AF-MSCs	Day 6	↑Graft renal function, ↓fibrosis	Baulier et al. [35]
2011	Rats	BM-MSCs	Three injections (day − 7, 0, 1)	↓Graft inflammation,	Hara et al. [36]

*MSCs* mesenchymal stem cells, *BM-MSCs* bone marrow MSCs, *BM-MSCs-MVs* MVs originated from bone marrow MSCs, *AF-MSCs* amniotic fluid-derived MSCs, *IDO* indoleamine 2, 3-dioxgenase, *DCs* dendritic cells, *Tregs* regulatory T cells, *AR* acute rejection, *NK cells* natural killer cells, *IF/TA* interstitial fibrosis/tubular atrophy

In addition to MSCs alone, microvesicles (MVs) derived from MSCs have also been regarded as a mediator of allograft tolerance in kidney transplantation. MVs are anuclear plasmalemmal vesicles that are formed by outward budding of the plasma membrane in a calcium- and calpain-dependent manner. MVs carry various biologically active substances, including lipids, proteins, enzymes, and coding and noncoding RNA molecules. Via secretion and endocytosis, these components can be transferred from one cell to another, acting in information exchange and inducing biological effects [37, 38]. The results obtained by Wu et al. suggested that MVs originating from bone marrow MSCs (BM-MSCs-MVs) were responsible for the prevention of AR in MV-treated mice. BM-MSCs-MVs dramatically enhanced the expression of miR-146a in DCs. Alteration of miR-146a inhibited its potential target gene Fas, and these effects together decreased the infiltration of DCs in transplanted tissues, favoring renal graft survival [28].

Based on these encouraging results, more studies with different timings, frequencies, or routes of MSC infusion have been performed. Merino et al. investigated two injections of the MSC regimen, which was given 4 days or 7 days before surgery, with a further infusion at day 0 in both groups. They confirmed that infusion either 4 days or 7 days before transplantation effectively decreased the percentage of T, B, and NK cells in peripheral blood, boosted the induction of Tregs, and prolonged graft survival. Furthermore, rats in the day − 7, 0 group presented with better creatinine levels, survival, and histological parameters than those in the day − 4, 0 group [29]. Similarly, a three injection regimen (days 0, 3, 7) conducted by Yu et al. and a four injection regimen (days − 7, 0, 7, 14) conducted by Zhang et al. both revealed protective effects of MSCs in a rat model of kidney transplantation [30, 31]. However, Casiraghi et al. reported an impact of the timing of MSC infusion on renal allograft survival and function. They demonstrated that in a sensitized mouse model of kidney transplantation, mice that received either a single (day − 7 or day − 1) or double (day − 7 and day − 1) pretransplantation injection of MSCs had better graft survival than untreated mice. In contrast, in mice that were given MSCs 2 days after transplantation, the infusion unexpectedly deteriorated renal graft function and caused AR [32]. In terms of delivery route, Zonta et al. found that only intraarterial infusion of MSCs was effective in the control of AR, whereas the intravenous approach was ineffective [33]. This evidence provides a more complicated view of the role of MSCs in kidney transplantation. There is no doubt that MSCs influence the immune system. However, some not yet fully explained negative factors might counteract their beneficial effects in alloimmunity.

In addition to the use of MSC infusion as an induction therapy, another aim of the application of MSCs in kidney transplantation is to improve chronic allograft nephropathy (CAN). Franquesa et al. established a rat model of CAN. In this model, the donated kidney is first exposed to a period of 2.5 h of cold ischemia and is then transplanted. Delayed MSC therapy was administered at 11 weeks postsurgery in the treatment group, and all rats were followed up to 24 weeks. As expected, at 24 weeks, the renal graft in the MSC group displayed normal histological alterations with minimal interstitial fibrosis/tubular atrophy (IF/TA) and cellular infiltration, in contrast with that in the control group. An immunohistochemical analysis showed an increased expression of IDO in the MSC group, indicating that the immunomodulatory properties of MSCs were dependent on IDO expression [34]. In addition to their immunoregulatory role, MSCs also present a strong regeneration capacity. In a porcine kidney autotransplantation model in which the rejection response is weak, the infusion of amniotic fluid-derived MSCs (AF-MSCs) at day 6 could lead to full renal function recovery and abrogated fibrosis development at 3 months [35]. Similarly, in a rat model of marginal kidney transplantation, Hara et al. found that a series of inflammatory factors, including TNF-α, IFN-γ, IL-1β, ICAM-1, CCL19, and CCL21, were downregulated after MSC infusion. They concluded that the administration of MSCs helped alleviate graft inflammation in the donated kidneys that had been kept at 4 °C for a prolonged period of 24 h. With this strategy, those organs formerly deemed untransplantable can be reutilized, which is meaningful for expanding the organ pool and overcoming the major obstacle of organ shortage in the field of kidney transplantation [36]. The evidence mentioned above suggests that MSCs are a generalist cell in the field of kidney transplantation that has the ability to induce different effects during treatment.

## Challenges encountered in clinical settings

Based on the exciting results from animal experiments, some clinical controlled trials were initiated (Table 2). A study by Tan et al. in 2013 was the first and largest randomized clinical trial (RCT) to date. They included three cohorts, with a total of 156 patients. The different therapeutic effects of MSCs compared with basiliximab were compared. In detail, 53 patients received MSCs followed by standard-dose CNIs, whereas another 52 patients received MSCs with low-dose CNI (80% of the standard dose) therapy. The remaining 51 patients were allocated into the control group and received basiliximab followed by a standard dose CNI regimen. After a 12-month follow-up, despite a reduced AR rate at 6 months and a decreased risk of viral infections in the two MSC groups compared with the control group, the delayed graft function (DGF) rate, renal graft function, and graft survival rate were all comparable between the MSC groups and the control group [10]. Another study by Pan et al. was a single-center, prospective, nonrandomized pilot study that was conducted in 2016. Infusion of MSCs with Cytoxan was compared with Cytoxan monotherapy in 32 patients. Consistent with the study by Tan et al., the combined MSC treatment only resulted in long-term CNI sparing, without additional renal graft benefits (as measured with levels of serum creatinine, urine protein, urinary RBCs, urinary WBCs, and AR rate) over a 24-month follow-up [11]. Similarly, the RCT by Sun et al. in 2018 also demonstrated that no differences in graft survival, DGF rate, AR rate, and renal graft function could be observed between groups of patients receiving either a combination of MSCs plus antithymocyte globulin (ATG) or ATG monotherapy [12]. Unlike the above trials, which all included injection of the first dose of MSCs on day 0, Erpicum et al. tried one injection of MSCs postsurgery at day 3 ± 2. However, except for the transient increase in the proportion of early Tregs seen, the MSC therapy did not provide benefits in terms of long-term effects, as measured with B cell frequencies, occurrence of opportunistic infections, renal graft function, graft survival rate, and AR rate [13]. Furthermore, due to safety reasons (NCT03585855) and deterioration of graft renal function after MSC infusion (NCT00752479), two trials were terminated. In these studies, it seemed that MSC treatment was not very advantageous over conventional regimens in clinical settings. Using key words such as “kidney transplantation” and “MSCs” to search related trials registered in ClinicalTrials.gov, some ongoing RCTs can be found. Of these, the Gaber group in Houston designed a dose-escalation RCT comparing the use of MSCs to saline administration in living donor kidney transplant recipients (NCT03478215). The Perico group in Bergamo is testing MSCs as a strategy to induce tolerance in kidney transplant recipients with a deceased donor (NCT03478215). These two trials are estimated to be completed in 2021. Considering the nephrotoxic side effects of CNIs, Reinders et al. are infusing MSCs into 35 kidney transplantation patients to facilitate tacrolimus withdrawal (NCT02057965). Another ongoing RCT is being conducted by a Chinese team, in which autologous stromal vascular fraction (SVF)-derived MSCs are being compared with basiliximab in terms of their ability to reduce posttransplant immunosuppressant use for recipients of living relative kidney transplantation (NCT02492308). These trials may provide us with a clearer view of the role of MSC-based therapy in kidney transplantation patients in the future (Table 3).

**Table 2:** Results from clinical control trials of MSCs in kidney transplantation

Author	Year	ClinicalTrials.gov identifier	Design of the study	Enrollment	MSCs type	MSCs doses	Timing of infusion	Inductions		Maintenance immunosuppressants	Positive outcomes	Negative outcomes
								Treatment group	Control group			
Tan et al. [10]	2013	NCT00658073	Single-center, prospective RCT	159	Autologous	Two injections (1–2 × 10^6^ cells/kg)	D0, D14	MSCs	Basiliximab	Steroids, MMF, 80% or standard dose of CNIs	Reduced AR rate at 6 months, decrease risk of viral infections	Comparable DGF rate, renal graft function, graft survival rate
Pan et al. [11]	2016	NM	Single-center, prospective, nonrandomized pilot study	32	Donor-derived	Two injections (5 × 10^6^, 2 × 10^6^ cells/kg)	D0, D30	MSCs + Cytoxan (200 mg/day D0–3)	Cytoxan (200 mg/day D0–3)	Steroids, MMF, 60% or standard dose of CNIs	CNI sparing	Comparable serum creatinine, urine protein, urinary RBC, urinary WBC, and AR rate
Sun et al. [12]	2018	NCT02490020	Multi-center prospective RCT	42	Allogeneic	Two injections (2 × 10^6^ cells/kg, 5 × 10^6^)	D0	MSC + ATG (50 mg/day D0–2)	ATG (50 mg/day D0–2)	Steroids, MMF, CNIs	Not observed	Comparable DGF rate, renal graft function, graft survival rate, and AR rate
Erpicum et al. [13]	2019	NCT01429038	Single-center, nonrandomized, controlled trial	20	Allogeneic	One injection (1.5–3 × 10^6^/kg)	D3 ± 2	MSCs+Basiliximab	Basiliximab	Steroids, antimetabolite, CNIs	Increased Tregs at D30	Comparable B cell frequencies, opportunistic infections, renal graft function, graft survival rate, and AR rate

*MSCs* mesenchymal stem cells, *NM* not mentioned, *RCT* randomized clinical trial, *ATG* antithymocyte globulin, *MMF* Mycophenolate mofetil, *CNIs* calcineurin inhibitors, *AR* acute rejection, *DGF* delayed graft function

**Table 3:** Registered clinical trials of MSC-based therapy in kidney transplantation according to ClinicalTrials.gov

ClinicalTrials.gov identifier	Aim of study	Enrollment	Phase	Status
NCT03478215	To investigate the safety and effectiveness of dose-escalation MSCs infusion compared to saline-only infusion in kidney transplantation	24	Phase 2	Recruiting
NCT02565459	To test MSCs as a strategy to induce tolerance in kidney transplant recipients	22	Phase 1	Recruiting
NCT02057965	To test the effectiveness of MSCs in combination with everolimus in facilitating tacrolimus withdrawal	70	Phase 2	Recruiting
NCT02492308	To determine the efficacy of autologous SVF derived MSCs in reduction of posttransplant immunosuppressants	120	Phase 1 and 2	Recruiting
NCT02409940	To evaluate the effect of allogeneic or autologous MSCs on immune cell response in kidney transplantation	17	Phase 1	Active but not recruiting
NCT02490020	To clarify the key role of MSCs to reduce AR and DGF after renal transplantation	260	Phase 1	Enrolling by invitation
NCT02561767	To determine the efficacy and safety of allogeneic MSCs in kidney transplantation	120	Phase 1 and 2	Not yet recruiting
NCT02563340	To investigate the efficacy and safety of allogeneic MSCs on chronic AMR after kidney transplantation	60	Phase 1 and 2	Not yet recruiting
NCT02563366	To investigate whether allogeneic MSCs can promote function recovery in patients with poor early graft function after kidney transplantation	120	Phase 1 and 2	Not yet recruiting
NCT03585855	To find out the effectiveness of MSCs in combination with standard therapy against AMR	4	Not applicable	Terminated (safety reason)
NCT00752479	To define the safety and biological/mechanistic effect of MSCs in living-related kidney transplant recipients	4	Phase 1 and 2	Terminated (necessity of major revision of the protocol)

*MSCs* mesenchymal stem cells, *SVF* stromal vascular fraction, *AR* acute rejection, *DGF* delayed graft function, *AMR* antibody-mediated rejection

## Promising outcomes with preconditioning strategies featuring MSCs in preclinical kidney transplantation studies

The evidence mentioned above indicates that there still exists a massive gap between preclinical observations and clinical applications. Impaired MSC function after infusion is thought to be the main reason for their limited effects. This is a common scenario when considering cell-based and cell-derived product therapies. Cell-based therapies must cope with interdonor variability and functional exhaustion [39]. After infusion, the fate and secretion profile of cells largely depend on the surrounding microenvironment. When in a harsh microenvironment caused by the pathological state, the regenerative capacity and secretome of transplanted cells will be damaged. Strong cues in a harsh microenvironment usually include hypoxia, heat, nutrient depletion, oxidative stress, inflammation, etc. [40, 41]. These factors can induce cell apoptosis and cause poor cell survival or impaired function of grafted cells [42]. Silva et al. concluded in their article that the limited clinical efficacy of cell-based therapy might be a result of poor engraftment, poor survival of cells, impaired regenerative ability, and delayed administration [43].

Similarly, the immunoregulatory and regenerative functions of MSCs in kidney transplantation largely rely on their efficient localization and secretion of factors within appropriate tissues. However, hypoxia, reactive oxygen species, and inflammatory reactions caused by kidney transplantation at the local site are major concerns that could impact the homing, proliferative, and secretory abilities of infused MSCs [44–46]. Kato et al. concluded in their article that “single administration of MSCs was insufficient to overcome the alloreactive T cell response totally and to achieve a long-term positive allograft outcome” [47]. Thus, the identification of novel strategies to enhance the properties of MSCs and maximize the effectiveness of MSC-based therapy is of great importance.

Based on these facts, some preconditioning methods have been explored. It is known that after injury, chemokines secreted by damaged cells can induce chemotaxis in nearby cells, recruiting them to the injured area and starting the regeneration process [48]. Some preconditioning methods were designed based on this theory. Stromal derived factor-1 (SDF-1) is thought to be upregulated at the local site due to the ischemic microenvironment after injury [49]. Meanwhile, its receptor, chemokine receptor 4 (CXCR4), was confirmed to exist on the surface of MSCs but is markedly reduced during ex vivo expansion [50]. Multiple studies have demonstrated that the regulation of the SDF-1/CXCR4 axis is an important mediator in the recruitment of MSCs to targeted tissues after injury. To assess whether overexpression of CXCR4 can improve the therapeutic effects of MSCs in kidney transplants, Cao et al. established CXCR4 gene-modified MSCs (CXCR4-MSCs) and injected them into rats undergoing kidney transplantation surgery. After 3 days, rats in the CXCR4-MSC group showed better renal function than rats in the control group, as assessed by both the serum creatinine level and pathological scores. In terms of the mechanism, the researchers found that the overexpression of CXCR4 helped promote homing of MSCs to the kidney graft. Moreover, a higher level of IL-10 and TGF-β was also observed in gene-modified rats versus control rats, which contributed to the higher percentage of CD25^+^Foxp3^+^ cells infiltrating the renal interstitium in the gene-modified rats [51]. Similarly, Zhang et al. tried to transfuse erythropoietin (EPO)-preconditioned MSCs (EPO-MSCs) into a rat kidney transplantation model. Increased expression of CXCR4 in MSCs was also successfully induced by preconditioning with EPO and was related to better survival and migratory ability. After transfusion, the administration of EPO-MSCs significantly ameliorated transplanted renal failure [52]. These results indicate that modulation of CXCR4 expression in MSCs not only was able to enhance MSC engraftment in the microenvironment after kidney transplantation but also helped improve their proliferative and secretory capacities, which induced better therapeutic effects in terms of graft renal function.

IDO is an important cytokine that plays a critical role in several immune cells, including T and B cells, APCs, and Tregs [53]. IDO can induce the peripheral conversion of T cells into Tregs, similarly to how it can affect MSC immunoregulatory actions. To investigate whether IDO and MSCs could induce a synergistic immunosuppressive effect in kidney transplants, He et al. transfected MSCs with the IDO gene (IDO-MSCs). In vitro, coculture of IDO-MSCs with peripheral blood mononuclear cells led to a greater decrease in the proportion of CD4^+^CD25^−^ effector T cells but induced a significantly higher percentage of CD4^+^CD25^+^Foxp3^+^ Tregs than incubation with control MSCs. In vivo, rabbits that received IDO-MSCs presented dramatically less AR, better graft renal function, and longer graft survival time than rabbits that received control MSCs. In a skin grafting test to examine tolerance in IDO-MSC rabbits, skin grafts from donor rabbits were shown to be well tolerated for over 60 days. Those grafts from nondonor or third-party rabbits were quickly rejected within almost a week. These findings revealed that via inhibition of allogeneic T cell proliferation and modulation of T cell subsets, IDO gene transfection enhanced the immune tolerance of MSCs in kidney transplants in a rabbit model [54]. In addition to IDO, the OX40-Ig fusion protein (OX40Ig) is another factor that can also induce T cell anergy and Treg transdifferentiation by blocking the OX40-OX40L pathway [55]. Liu et al. synthesized OX40Ig gene-modified MSCs (OX40Ig-MSCs). Similarly, rats that received OX40Ig-MSCs presented better graft renal function and longer graft survival time than those in the wild-type MSC group; the rats that received OX40Ig-MSCs also showed less AR, lower levels of IFN-γ, and upregulated expression of IL-10, TGF-β, and Foxp3, indicating OX40Ig-MSCs were advantageous over control MSCs in the prevention of graft rejection, improvement of graft function, and prolongation of graft survival [56] (Table 4).

**Table 4:** Promising outcomes with preconditioning strategies on MSCs in preclinical kidney transplantation studies

Year	Animal	MSCs source	Preconditioning	Timing of infusion	Outcomes	References
2013	Rats	BM-MSCs	Gene modification	Day 1	↑CXCR4, ↑proliferative, secretory and migratory ability, ↑Tregs, ↓pathological scores, ↑graft renal function	Cao et al. [51]
2018	Rats	BM-MSCs	Incubation with trophic factors/cytokines	Day 0	↑CXCR4, ↑survival and migratory ability, ↓pathological scores, ↑graft renal function	Zhang et al. [52]
2015	Rabbits	BM-MSCs	Gene modification	Day 0	↑IDO, ↑Tregs, ↓T cells, ↓AR, ↑graft renal function and survival	He et al. [54]
2016	Rats	A-MSCs	Gene modification	Day − 4	↑OX40Ig, ↑Tregs, ↓AR, ↑graft renal function and survival	Liu et al. [56]

*MSCs* mesenchymal stem cells, *BM-MSCs* bone marrow MSCs, *A-MSCs* adipose MSCs, *CXCR4* chemokine receptor 4, *EPO* erythropoietin, *IDO* indoleamine 2, 3-dioxgenase, *OX40Ig* OX40-Ig fusion protein

In addition to enhancing the ability to modulate the immune system, another advantage of applying a preconditioning strategy to MSCs in kidney transplantation is improving the capacity of the transplanted kidney to withstand injury. This is particularly important in the revival of graft renal function after transplantation, considering that I/R injury is an inevitable process during surgery. The ability of MSCs to increase therapeutic effects has been verified in various kinds of I/R injury-induced diseases, including acute kidney injury (AKI) [57], acute myocardial infarction [58], and ischemic stroke [59]. Taking I/R-induced AKI (I/R-AKI) as an example, similar to the situation in kidney transplantation, the mechanisms underlying the enhanced protective effects gained with preconditioning of MSCs in AKI are also based on the improved function of infused cells. Via multiple signaling pathways, different preconditioning strategies can enhance the homing, proliferative, and secretory capacities of MSCs in vivo, inducing tissue repair functions. Liu et al. identified that preconditioning MSCs with EPO could significantly increase the chemotactic migration of transplanted MSCs into the ischemic kidney. Local upregulation of SDF-1 levels and activation of the PI3K/AKT and MAPK signaling pathways in MSCs might account for the enhanced beneficial effects [60]. Masoud et al. found that after incubation with S-nitroso N-acetyl penicillamine, which is an NO donor, the expression of AKT increased several-fold in MSCs. Alterations in AKT levels further promoted MSC survival, proliferation, and growth [61, 62]. Regarding secretory capacity, many studies have demonstrated that pre-exposure of MSCs to hypoxia induces increased levels of fibroblast growth factor and vascular endothelial growth factor and a better therapeutic outcome in preclinical I/R-AKI models. A suitable preconditioning method is necessary not only to assist MSCs in better modulating the immune response but also to enable their tissue regeneration capacity, which together contribute to inducing long-term tolerance of the graft.

## Conclusions and future perspectives

MSCs have shown beneficial effects in the treatment of animal kidney transplantation models. However, difficulties with poor survival, engraftment, secretion, and differentiation have limited the therapeutic effects of simple MSC transplantation in clinical settings. Research on the preconditioning of MSCs has revealed that this strategy has potential advantages over conventional MSC infusion, opening new doors for clinical applications.

However, despite the promising future of this strategy, some issues need to be solved before successful translation into clinical settings. First, exogenous infusion of cell products bears the risk of transfusion reactions, such as allergic reactions, fever, and hypotension. Embolisms should also be monitored for if a large dose is administered. In addition, there are specific preconditioning strategies that require more caution than others. Hypoxia preconditioning and preconditioning with physical factors are relatively safe, with few adverse effects. However, incubation with pharmacological/chemical agents or trophic factors/cytokines or preconditioning with gene modification strategies theoretically increases the potential to enhance tumor formation and progression. Although no studies have reported the development of de novo malignancy after preconditioned MSC injection to date, the risks and advantages should be considered. Second, the risk of infection needs to be considered. Data from clinical studies vary with respect to the infection rate after infusion with MSCs in kidney transplantation patients. Due to their immunosuppressive effect, traditionally, it was thought that the concomitant use of MSCs with immunosuppressive medications would increase the rate of infection, which was also confirmed in the study by Reinders et al [63] However, Tan et al. demonstrated contrasting results [10]. This issue should be closely monitored in future studies. Third, the risk of allograft dysfunction needs to be considered. Evidence from both preclinical and clinical studies suggests that MSCs can acquire a proinflammatory phenotype in some circumstances. The reason is still not clear, but the specific timing of infusion could be an important factor. Fourth, immunogenicity needs to be considered. Although the traditional view regards MSCs as nonimmunogenic cells due to the absence of MHC II molecules on their surface, concern regarding the immune reaction activated by transfused MSCs or the production of donor-specific antibodies still exists. In addition, the long harvesting period and constantly impaired cell function due to the urotoxic environment in recipients restrict the clinical application of autologous MSCs. Fifth, the cost needs to be considered. The lack of a standardized process and the complex incubation techniques make MSC treatment a costly regimen. In addition, such an expensive treatment provides only a marginal beneficial effect. There is an urgent need to establish a standard for MSC production and achieve large-scale commercialization as soon as possible. Last, is preconditioning the best strategy to overcome the low efficacy of MSCs clinically? Potential strategies to enhance the therapeutic potency of MSCs include optimization of the source (allogeneic or autologous, bone marrow, adipose or umbilical cord sources), route (intravenous, intraarterial or local administration), timing (presurgery or postsurgery), and frequency (single injection or multiple injections) of MSC therapies. Studies comparing different strategies are lacking. However, due to the always neutral or sometimes contradictory results obtained in applying those strategies, preconditioning is a definite method that can indeed enhance the therapeutic effects of MSCs. Therefore, in our opinion, preconditioning is the most promising way to solve the issues mentioned above. These issues can in part explain why available therapeutic regimens with MSCs in the field of kidney transplantation remain scarce. Much work still needs to be done in the future.

In conclusion, we look forward to a future in which MSC preconditioning can be used to achieve long-term benefits in kidney transplantation patients. At the same time, caution should also be applied when designing future studies. With further fine-tuning of aspects such as infusion timing and route, concurrent immunosuppressive treatment, and cotreatments, the full immunomodulatory properties of MSCs can be exploited, enabling MSCs to become a powerful cell therapy in clinical kidney transplantation.

## Data Availability

Not applicable.

## Abbreviations

MSCs: Mesenchymal stem cells
ESRD: End-stage renal disease
CNIs: Calcineurin inhibitors
AR: Acute rejection
MHC: Major histocompatibility complex
I/R: Ischemia/reperfusion
Tregs: Regulatory T cells
APCs: Antigen-presenting cells
DCs: Dendritic cells
NK cells: Natural killer cells
IDO: Indoleamine 2, 3-dioxgenase
MVs: Microvesicles
BM-MSCs-MVs: MVs originated from bone marrow MSCs
CAN: Chronic allograft nephropathy
IF/TA: Interstitial fibrosis/tubular atrophy
AF-MSCs: Amniotic fluid-derived MSCs
RCT: Randomized clinical trial
DGF: Delayed graft function
ATG: Antithymocyte globulin
SVF: Stromal vascular fraction
SDF-1: Stromal derived factor-1
CXCR4: Chemokine receptor 4
CXCR4-MSCs: CXCR4 gene modification MSCs
EPO: Erythropoietin
EPO-MSCs: EPO preconditioning MSCs
IDO-MSCs: MSCs with IDO gene
OX40Ig: OX40-Ig fusion protein
OX40Ig-MSCs: OX40Ig gene-modified MSCs
AKI: Acute kidney injury
I/R-AKI: I/R induced AKI
